# Obstetric anesthesia/analgesia does not affect disease course in multiple sclerosis: 10‐year retrospective cohort study

**DOI:** 10.1002/brb3.1082

**Published:** 2018-07-25

**Authors:** Hana Harazim, Pavel Štourač, Petr Janků, Hana Zelinková, Kamil Frank, Michal Dufek, Petr Štourač

**Affiliations:** ^1^ Department of Anaesthesiology and Intensive Care Medicine Medical Faculty of Masaryk University University Hospital Brno Brno Czech Republic; ^2^ Department of Neurology Medical Faculty of Masaryk University University Hospital Brno Brno Czech Republic; ^3^ Department of Gynaecology and Obstetrics Medical Faculty of Masaryk University University Hospital Brno Brno Czech Republic; ^4^ Institute of Biostatistics and Analysis Medical Faculty of Masaryk University Brno Czech Republic; ^5^ First Department of Neurology Medical Faculty of Masaryk University St Anne's University Hospital Brno Czech Republic; ^6^ Department of Paediatric Anaesthesiology and Intensive Care Medicine Medical Faculty of Masaryk University University Hospital Brno Brno Czech Republic

**Keywords:** cesarean section, labor, multiple sclerosis, obstetric anesthesia, pregnancy, relapse

## Abstract

**Objectives:**

Multiple sclerosis (MS) often occurs in young women and the effect of obstetric anesthesia/analgesia on the disease is poorly understood. No previous study has investigated the course of the disease in women in labor in the Czech Republic. The aim of this study was to evaluate the occurrence or absence of relapses in the 6‐month postpartum period in MS parturients with and without obstetric anesthesia/analgesia.

**Materials and Methods:**

We retrospectively studied all deliveries (*n* = 58,455) at the University Hospital Brno from 2004 to 2013 and identified those of the women with an ICD‐10 code G35 (MS) recorded anytime in their medical history (*n* = 428). We included only deliveries of women with confirmed diagnosis at the time of labor (*n* = 70). Statistical analysis was performed using the Fischer Exact Test.

**Results:**

There were 70 deliveries of 65 women, including 45 vaginal deliveries and 25 Cesarean deliveries (16 under general anesthesia, 8 with epidural anesthesia and 1 with spinal anesthesia). Epidural obstetric analgesia was performed in 11 deliveries. There was no statistically significant difference in relapses between the vaginal delivery group (*n* = 15; 33%) and Cesarean section group (*n* = 10; 40%), *p* = 0.611.

**Conclusion:**

Neither delivery mode (vaginal vs Caesarean) nor type of obstetric anesthesia/analgesia was found to have any impact on the course of MS at 6 months postpartum in women with this condition.

## 1 INTRODUCTION

The prevalence of multiple sclerosis (MS) is 160 per 100,000 individuals in the Czech Republic (Vachova, 2012) and affects women two to three times more than men (Sellner et al., 2011). There is a difference in the incidence and prevalence of MS across Europe with suspected risk factors presumed to be a combination of lack of vitamin D, smoking and EBV infection together with genetic predisposition for MS (Vachova, 2012). One recent meta‐analysis detected significant heterogeneity from Hungary to Saskatchewan for familial MS prevalence that was not latitude or ethnicity dependent, highlighting the accumulative effects of genetic and environment factors on its prevalence (Harirchian, Fatehi, Sarraf, Honarvar, & Bitarafan, 2018).

Multiple sclerosis often manifests in early adulthood (Compston & Coles, 2002) with an average onset age of 32 years, (Shirani, Zhao, Kingwell, Rieckmann, & Tremlett, 2012) making pregnancy issues of real importance in women with MS. Studies have shown that between 1/5 and 1/3 of women with MS bear children after disease onset, (Runmarker & Andersen, 1995; Weinshenker, Hader, Carriere, Baskerville, & Ebers, 1989) making the effect of maternal MS on pregnancy outcomes relevant to patients, their family members, and health care professionals (van der Kop et al., 2011). In the past, pregnancy was discouraged in women with MS; however recent studies have shown pregnancy as having a potentially beneficial role on MS relapse rates with no effects on long‐term progression of the disease (Confavreux, Hutchinson, Hours, Cortinovis‐Tourniaire, & Moreau, 1998; Koch, Uyttenboogaart, Heersema, Steen, & De Keyser, 2009; Vukusic et al., 2004).

Recommendations on labor management in MS women available in the Czech Republic are calling for extra consideration when vaginal labor carries a high risk of parturient exhaustion. Indications for Cesarean section (C‐section) is strictly obstetric, vaginal delivery is recommended. Methods of obstetric analgesia should be available to the patient, including epidural obstetric analgesia (EOA) (Houtchens, 2013).

The opinion of anesthesiologists in the Czech Republic on regional anesthesia (RA) in MS patients was notably formed by Larsen′s Anesthesia, a worldwide well‐respected textbook which states: “Brain and spinal diseases are generally considered to be absolute contraindications for subarachnoid anesthesia, especially if these diseases are still florid, as in the case with MS. The reason for contraindication is rather forensic, because it is necessary to avoid a situation where the patient exchanges a random worsening of the neurological condition for a subarachnoid damage. Contraindications to epidural analgesia are consistent with spinal anesthesia, relative contraindications are some neurological disorders” (Larsen, 2004).

Despite initial concerns that pain management techniques could worsen the short (or long) term course of MS, existing studies have shown no effect on overall MS disability (Confavreux et al., 1998; Pasto et al., 2012) or the precipitation of MS relapse (Confavreux et al., 1998; Dalmas, Texier, Ducloy‐Bouthors, & Krivosic‐Horber, 2003; Pasto et al., 2012; Vukusic et al., 2004).

The aim of this retrospective study was to describe relapse occurrence (RO) in parturients with MS at a 6‐month postpartum interval depending on the presence of obstetric anesthesia and/or analgesia. We were working with the null hypothesis that there was no difference in MS course depending on type of anesthesia/analgesia used.

## 2 MATERIALS AND METHODS

### 2.1 Study design

This retrospective cohort study was conducted at a tertiary care University Hospital Brno with average of 6,000 births per year. The study was approved by the Ethics Committee of the University Hospital Brno in Brno (July 24, 2014) with a waiver to Informed Consent. The study was registered at ClinicalTrials.gov (ID: NCT03247894).

The primary outcome was to describe relapse occurrence in MS patients depending on the type of labor (vaginal or C‐section) at 6‐month postpartum.

The secondary outcome was to evaluate possible influence of the different anesthesia/analgesia types on the course of MS. RO in parturients who delivered via C‐section with general anesthesia (GA) compared to C‐section with RA; and parturients delivering vaginally with EOA compared to labors without EOA. We also looked at RO in the 3‐month postpartum interval in groups of vaginal delivery and C‐section.

### 2.2 Study participants

We aimed to enroll all parturients with MS who met the inclusion criteria: MS was confirmed before labor Poser et al. (1983) or McDonald et al. (2001); Polman et al. (2011) criteria and we were able to detect course of the disease. Parturients without proper neurological follow‐up before labor and in the 6‐month interval after labor were excluded. For the purpose of this study, data collected in years 2004–2013 were used.

For identification of the study group, we used the medical records of University Hospital Brno. First, we created a list of patients who were recorded from January 1 2004 to December 31 2013 as having a diagnostic code O80‐O84 meaning different types of birth (International Classification of Diseases, ICD‐10). We then matched this with a list of all patients who had at least once in their history a recorded code G35 for MS (ICD‐10). Hospital labor records of all patients matching both lists were taken from the hospital archives and scanned for the presence of MS in the personal anamnesis. Births to mothers whose disease onset occurred after delivery were excluded. Records of patients with an invalid use of the G35 code and those with unconfirmed MS at the time of labor were also excluded. Clinical and therapeutic data were collected from hospital records of labor by the anesthesiologist and gynecologist. The presence of MS in all parturients was confirmed by a neurologist from the Multiple Sclerosis Centre of the University Hospital Brno using their medical records. The neurologist collected data on the status of the disease before delivery and two trimesters after labor. When a patient was observed in another MS center, we contacted their specialists to collect the data from their medical records.

### 2.3 Observed parameters

For each labor, we recorded the following data (Table 1).

**Table 1:** Observed parameters

General characteristics	Neurologic data		Anesthesiologic data			Obstetric data	Neonatal data
Maternal age (yrs)	Date of MS onset		ASA status			Pregnancy	Gestation age at delivery
Maternal prepregnancy weight (kg)	Age at MS onset		Number of previous anesthesias			Parity	Gender
Maternal weight at delivery (kg)	MS Duration		Type of anesthesia for delivery			Singleton or multiple pregnancy	Weight (g)
Maternal height (cm)	Number of relapses from MS onset to labor		Repetition of anesthesia in 48 hr after delivery			Pregnancy complications	Length (cm)
Maternal BMI (kg/m²)	Type of treatment before study pregnancy		**Management of GA**	**Management of RA**	**Management of EOA**	Antenatal steroids for pulmonary maturation	Apgar score ‐ 1 min
Additional morbidity	Need for therapy escalation during pregnancy		Induction agent	Type of LA	Type of LA	Complication of delivery	Apgar score ‐ 5 min
Date of Labor	Extent of neurologic disability before pregnancy		Muscle relaxant	Volume and concentration of LA	Volume and concentration of LA	C‐section duration (min)	Apgar score ‐ 10 min
Length of hospital stay during pregnancy (days)	**Relapse Occurrence in 3 months postpartum**	**Relapse Occurrence in 6 months postpartum**	Opioid	Needle gauge	Needle gauge	Blood loss (ml)	Turbidity of amniotic fluid
Length of hospital stay for delivery (days)	Number of new relapses	Number of new relapses	Volatile anesthetics	Position of puncture	Position of puncture	Placental adhesion disorder	CTG abnormalities
Type of delivery (vaginal/C‐section)	New treatment	New treatment	Muscle relaxant reversal agent	Complication of puncture	Complication of puncture	Blood products transfusion (in 72 hr after delivery)	Cord Blood Gas Analysis (pH)
Type of C‐section (elective/urgent)	New MRI lesions	New MRI lesions		Administration of antihypotensives	Administration of antihypotensives	Lactation suppression (indication, day after delivery)	Length of neonatal ICU stay
Indication of C‐section				Insertion of epidural catheter	Insertion of epidural catheter	Puerperal morbidity	Congenital developmental defect

Note. BMI: body mass index; CTG: cardiotocography; EOA: epidural obstetric analgesia; GA: general anesthesia; ICU, intensive care unit; LA: local anesthetic; RA: regional anesthesia.

The bold fonts inside the table are for subheadings. Under these subheadings are observed parameters of this segment of examination.

Relapse occurrence (RO) in the 6‐month period after delivery was recorded (we also recorded relapses in the first 3 months after delivery). A relapse was defined as the appearance or worsening of symptoms of neurologic dysfunction lasting more than 24 hr, new lesions on an MRI or need of reinforced treatment. For disease status after delivery, we used dichotomic approach: relapse (at least one) or no relapses.

### 2.4 Methods of anesthetic management for GA, RA and EOA

The term analgesia is used to define pain management for labor, whereas anesthesia refers to pain management for an interventional procedure (such as C‐section) (Youngstrom, Baker, & Miller, 1996). While recognizing that epidural anesthesia and spinal anesthesia are not interchangeable terms, for simplicity, the term RA will be used to describe both techniques. The obstetric analgesia was delivered either in the form of epidural analgesia or as intravenous remifentanil using a parturient‐controlled analgesia device (Stourac et al., 2014). No other anesthetic methods for labor pain relief were used.

We referred to the annual labor and neonatal statistics of the University Hospital Brno from the year 2013 to compare the relative frequency of delivery types in common parturient populations with our group of MS women (Hruban, 2014).

The potential sources of bias were relatively minor, selected and retrospective sample of participants.

The following covariates were assumed possible confounders: age, parity (nullipara vs multipara), birth weight, age at MS onset, age, disease duration, DMDs before pregnancy, and need for reinforcement of MS therapy during pregnancy.

### 2.5 Statistics

The analysis of association between presence of anesthesia/analgesia and occurrence of relapses in two intervals (3 and 6 months after birth) was performed. We grouped cases by mode of delivery: assisted vaginal deliveries (use of forceps or vacuum extractor) and spontaneous vaginal deliveries; C‐section in RA. As for obstetric analgesia use, we analyzed vaginal deliveries with EOA and vaginal deliveries without EOA, these included a subgroup of labors with administered rPCA.

Contingency was assessed with the Fischer two‐tailed exact test. All statistical analyses were conducted using Statistica 12, StatSoft Inc., http://scicrunch.org/resolver/SCR_014213. The sample size was not estimated a‐priori. A *p* value equal toor less than 0.05 was considered statistically significant. All labors with unknown neurological follow‐up were excluded from analysis.

## 3 RESULTS

### 3.1 Characteristics of the women with MS

**Figure 1:**
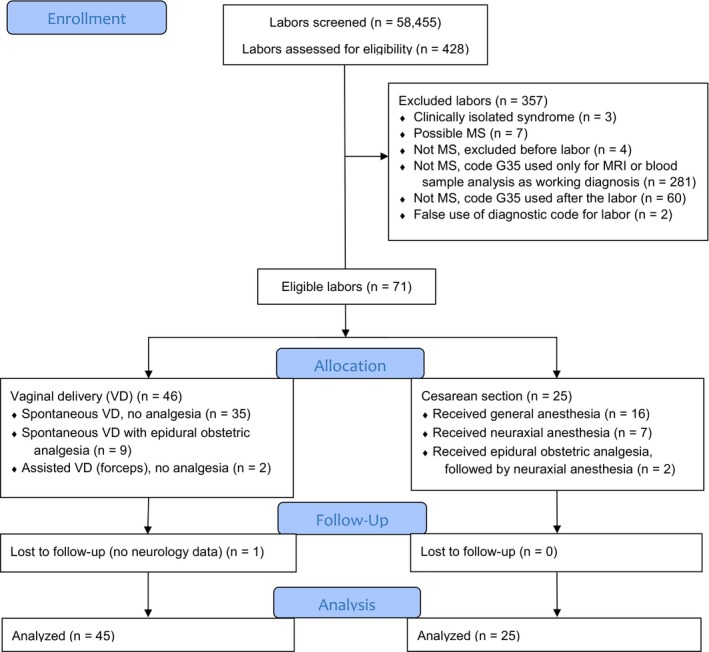
Flow diagram illustrating patient recruitment into the study

Between August 2014 and May 2017, we screened 58,455 labors and identified 471 hospitalization records, matching 428 deliveries of 340 women. The recruitment is pictured in Figure 1.

Full data on pregnancy, delivery, anesthetic management, and neurologic outcome were available for all 70 pregnancies. The base‐line characteristics of parturients are shown in Table 2, we found no statistically significant differences between groups of vaginal delivery and C‐section. All parturients suffered from relapsing‐remitting form of MS, 24 of them were without any treatment for MS before pregnancy, four patients required fortification of MS therapy during pregnancy.

**Table 2:** Study sample characteristics

	Vaginal delivery (*n* = 45)		Cesarean section (*n* = 25)	
	Mean (*SD*)	Median (Min‐Max)	Mean (*SD*)	Median (Min‐Max)
Maternal age (years)	28.5 (4.1)	29 (18–38)	30.6 (4.4)	30 (22–41)
Age at MS onset (years)	22.9 (5.1)	22 (14–38)	24.5 (4.8)	25 (15–33)
Duration of MS (years)	5.6 (4.2)	5 (0–20)	6 (4.0)	6 (0–15)
Maternal weight at delivery (kg)	77 (13.5)	76 (57–124)	75.3 (14.8)	76 (56–121)
Maternal height (cm)	168 (5.3)	168 (158–180)	167.6 (5.5)	167 (160–180)
Maternal BMI at delivery (kg/m²)	27.4 (4.8)	27 (20.2–43.9)	26.9 (5.1)	26 (19.8–44)
Gestational age at delivery (weeks)	38.8 (1.6)	39 (34–41)	38.5 (1.0)	38 (36–41)
Number of relapses from MS onset to labor	2.5 (2.2)	2 (0–10)	2.7 (1.7)	2 (1–6)

	Count	Incidence	Count	Incidence
DMDs before pregnancy (yes)	30	67%	14	56%
Gravidity				
I	28	62%	11	44%
II	10	22%	10	40%
III	4	9%	4	16%
IV	3	7%	0	0%
Parity				
Nulliparous	32	71%	13	52%
Multiparous	13	29%	12	48%
ASA				
I	3	7%	0	0%
II	42	93%	24	96%
III	0	0%	1	4%
IV	0	0%	0	0%

Note. ASA: American Society of Anesthesiologists Physical Status; BMI: Body Mass Index; DMDs: Disease‐Modifying Drugs.

### 3.2 Primary outcome results

We recorded RO in vaginal delivery and C‐section groups. In the former, (*n* = 45) 10 women (22%), had a relapse in 0–3 months in 4–6 months, six women (13%) and overall RO in 0–6 months in 15 women (33%). In the C‐section group (*n* = 25) there was RO in 0–3 months in eight women (32%), in 4–6 months, in seven women (28%), and in 0–6 months in 10 women (40%). No statistically significant difference between the groups was found in any interval (0–3 months, 4–6 months, 0–6 months; *p* values: 0.4028, 0.1987, 0.6106, respectively) and we accepted the null hypothesis.

### 3.3 Secondary outcome results

In the full study cohort, we reported experience of relapse in 25.7% of cases during the first trimester postpartum, in 18.6% of cases during the second trimester postpartum, with overall relapse in 35.7% cases in 6 months after delivery. Thirty‐four women received anesthesia or analgesia but the differences in the RO at 6 months after delivery were insignificant.

Differences in the RO at 6 months after delivery between groups with and without EOA were insignificant. There were 45 vaginal deliveries, including two assisted vaginal labors using forceps. EOA was given to 11 women; 10 of whom were nulliparous, two of the labors terminated in C‐section. Of nine parturients with EOA who delivered vaginally, two experienced relapse (22%) at 6 months after labor. In 36 parturients without EOA, who delivered vaginally, there was RO in 13 cases (36%). Remifentanil parturient‐controlled analgesia was administered in two women.

There were no significant differences in RO 6 months after delivery between groups with C‐section in GA and C‐section in RA. In the C‐section group, there were 16 general (64%), eight epidural (32%), and one spinal anesthesia (4%). In the group of GA, there was relapse in seven cases (44%). Relapses occurred in the group of RA in three cases (33%).

C‐section rate differed for nulliparous (28%; 13 of 45) and multiparous group (48%; 12 out of 25); multiparous women were more likely to deliver via C‐section. The indication of CS was recorded as neurological due to MS in 18 deliveries, in six cases it was obstetric indication (hypoxia, dystocia, breech delivery).

Neonatal characteristics showed no significant differences (Table 3). Nonsingleton births and nonlive births were not present. No congenital defects of newborns were present. Only one infant was recorded in 5 min with Apgar score lower than 7 (4‐6‐6). Pharmacological lactation suppression was performed in 9 cases, all indications stated as neurological.

**Table 3:** Neonatal characteristics

	Vaginal delivery (*n* = 45)		Cesarean section (*n* = 25)	
	Mean (*SD*)	Median (Min–Max)	Mean (*SD*)	Median (Min–Max)
Length (cm)	49.5 (2.3)	50 (43–56)	48.6 (2.2)	49 (44–52)
Weight (g)	3,297 (492)	3,300 (2,100–4,560)	3,147 (384.4)	3,100 (2,440–4,030)
Blood pH	7.28 (0.1)	7.27 (7.11–7.47)	7.28 (0.05)	7.28 (7.16–7.35)
Apgar 1st min	8.8 (1.2)	9 (4–10)	8.6 (1.1)	9 (5–10)
Apgar 5th min	9.5 (0.8)	10 (6–10)	9.4 (0.6)	9 (8–10)
Apgar 10th min	9.7 (0.7)	10 (6–10)	9.8 (0.5)	10 (8–10)
Gestational age (weeks)	38.8 (1.6)	39 (34–41)	38.5 (1.0)	38 (36–41)

	Count	Incidence	Count	Incidence
Gestational age				
24–36 weeks	5	11%	1	4%
37–40 weeks	36	80%	23	92%
>40 weeks	4	9%	1	4%
Gender (male)	23	51%	13	52%
Turbidity of amniotic fluid (turbid)	3	7%	2	8%

## 4 DISCUSSION

The most important finding of our study was that relapse after labor does not depend on either type of delivery or type of anesthesia for C‐section (CS). There was also no impact of epidural analgesia on relapse. This corresponds with the outcome of the PRIMS study (254 women with MS, C‐sections 43, EOA 42) (Confavreux et al., 1998; Vukusic et al., 2004) showing no significant difference in the rate of relapse between women who underwent epidural analgesia and those who did not. However, this was a secondary outcome of the study and no detailed analysis was provided (Confavreux et al., 1998). Another analysis of a cohort used to determine pregnancy and fetal outcomes in MS patients with interferone (CS 155, EA 65) also showed no correlation between EOA, CS and postpartum relapse (Pasto et al., 2012). One small prospective study of 19 women (EOA 10, CS 1) concluded that EOA was innocuous in the context of MS patients (Dalmas et al., 2003). There are no other recent studies regarding type of anesthesia for delivery in MS patients.

Our study results of postpartum relapses are consistent with data in other studies (Finkelsztejn, Brooks, Paschoal, & Fragoso, 2011; Vukusic et al., 2004) showing the largest number of relapses in the first trimester postpartum.

There was controversy in the past on the safe use of RA in patients with MS, but fears about increased relapse rate following RA were fortunately not borne out in a large prospective series (Confavreux et al., 1998). Nevertheless, many anesthetists still believe that GA causes fewer exacerbations than neuraxial blocks, particularly spinal anesthesia (Dalmas et al., 2003). The background to these concerns on the use of RA in MS patients was established by small retrospective study by Bader in 1988 (32 deliveries, CS 8, EOA 9). Spinal anesthesia has been implicated in postoperative exacerbation of the disease, presumably due to the increased susceptibility of demyelinated neurons to the neurotoxic effects of local anesthetics (Bader, Hunt, Datta, Naulty, & Ostheimer, 1988; Bamford, Sibley, & Laguna, 1978). However, there is a dearth of definitive studies on the pharmacologic effects of local anesthetics used in neuraxial anesthesia in women with MS and initial studies showing a higher risk of relapse in a patient who underwent spinal anesthesia have not been duplicated (Lirk, Birmingham, & Hogan, 2011).

In contrast with these earlier findings, we found no evidence of higher occurrence of relapses in women with CS under RA compared to CS under GA. These results support the idea that epidural anesthesia seems to involve less risk because local anesthetic reaches the intrathecal space in lower concentrations than intrathecal administration in spinal anesthesia (Stoelting & Dierdorf, 1993). Recommendations have been made to limit neuraxial dosing to the lowest level to achieve adequate pain management in all patients during labor and birth (Lirk et al., 2011) and to avoid paresthesia or epinephrine (Vercauteren & Heytens, 2007). Local anesthetics may directly interact with MS lesions and temporarily worsen symptoms by blocking sodium channels in demyelinated spinal areas (Lirk et al., 2011). However, any stressful condition, fever/hyperpyrexia, infection, surgery, delivery, and fatigue may cause exacerbations or relapses, making it very difficult to separate the effects of these factors and anesthetic techniques or drugs used (Korn‐Lubetzki, Kahana, Cooper, & Abramsky, 1984). Most authorities therefore consider that RA should not to be contraindicated in these patients, and many recommend its use in labor to reduce fatigue (Reide & Yentis, 2010).

In 2013 at the University Hospital Brno, there were 6,053 babies born and 5,902 labors, including 21.1% C‐sections, 4.1% assisted vaginal deliveries, and 28.7% parturients receiving EOA (Hruban, 2014). The rate of GA for CS in the hospital was described as 29.5% in 2014 (Stourac et al., 2015). In our study group, we reported 35% C‐sections, 2.85% assisted vaginal deliveries, and 15.7% parturients received EOA. Interestingly, there was a significant difference in the frequency of CS in MS patients (35%) and current parturients (21.1%), raising questions regarding indications for CS in these patients. We reported six cases of obstetric indications in 25 cases of CS and another 18 cases as neurological indications due to MS. One literature review and meta‐analysis showed possible higher prevalence of C‐sections (between 9.6% and 41.10%) as a reflection of cultural, religious, geographical and regional backgrounds (Finkelsztejn et al., 2011). Another remarkable finding is the rate of GA for CS in the MS group (64%) that is more than twice as high asthe routine CS rate in the University Hospital Brno (29.5%) (Stourac et al., 2015). The GA rate for an obstetric patient in this investigation exceeds by far that reported by Drake et al. in the United Kingdom. In their questionnaire given to 592 anesthesiologists on obstetric regional blocks for MS women, RA vastly dominated; 2% would prefer GA for elective CS and 3% would give GA for emergency CS with time only for single‐shot spinal (Drake, Drake, Bird, & Russell, 2006).

The main strength of the present work was the consistent, descriptive, and representative sample of parturients with MS. The University Hospital Brno, where we identified all patients in labor with confirmed MS over a period of 10 years, is the largest perinatological center in the Czech Republic. We successfully gathered data and analyzed 70/71 deliveries, with only one woman lost to follow‐up.

The main limitation is its retrospective character, making it difficult to acquire accurate data on the neurological status, postpartum. Prospective randomized double‐blind study on the use of obstetric anesthesia in MS patients are, in any case incompatible with medical ethics standards. The other limitation of the study is the patient's refusal of regional blockade (for C‐section or obstetric analgesia) which is regarded as a main contraindication of these techniques. Patients with a more serious disease status may tend to avoid regional blockade. There may be other reasons why epidural anesthesia use differs in women with MS and the general population, e.g. parity or disease duration (Lu et al., 2013).

Antenatal counseling and consultation between patient, obstetrician, neurologist, and anesthetist are advocated in order to create a management plan regarding the birth and analgesic options. It is particularly important to discuss the possibility of a regional anesthetic technique, accurately document any pre‐existing neurological deficit (Hinova & Fernando, 2010) and not refuse appropriate anesthetic management on the grounds of medico‐legal concerns (Warren & Fletcher, 1986). Recent data show that increased postnatal relapse rates seem to be the only real risk for these women (Finkelsztejn et al., 2011), irrespective of the type of anesthesia (Hinova & Fernando, 2010). Anesthetists should not automatically take all the responsibility in the case of progressive or new deficits after the procedure (Vercauteren & Heytens, 2007).

## 5 CONCLUSION

This study confirmed no difference in postpartum MS course, regardless of type of delivery, type of anesthesia for C‐section and use of EOA. Large prospective trials could provide more definitive evidence in this subject.

## CONFLICT OF INTEREST

The Authors declare that there is no conflict of interest.
